# Controlled Blood Pressure in Iranian Patients: A Multi-Center Report

**DOI:** 10.5539/gjhs.v8n4p188

**Published:** 2015-08-18

**Authors:** Ezzatollah Sadeghi, Azin Behnood-Rod, Hossein Aerab-Sheibani, Elham Shobeiri, Pirouz Pourzargar, Ehsan Ormoz, Nader Sadigh, Yashar Moharamzad

**Affiliations:** 1 Department of Surgery, School of Medicine, Kermanshah University of Medical Sciences, Kermanshah, Iran; 2 Islamic Azad University, Tehran Medical Branch, Tehran, Iran; 3 Shahid Sadoughi University of Medical Sciences, Bafgh, Yazd, Iran; 4 School of Medicine, Kermanshah University of Medical Sciences, Kermanshah, Iran; 5 Cardiovascular Research Center, Shahid Beheshti University of Medical Sciences, Tehran, Iran; 6 Department of Statistics, Mashhad Branch, Islamic Azad University, Mashhad, Iran; 7 Medical Research Supervisor, Sharif Entrepreneurs and Researchers Institute, Tehran, Iran

**Keywords:** hypertension, blood pressure, control, Iran

## Abstract

We decided to determine the percentage of hypertensive patients whose blood pressure (BP) measurements were within recommended controlled range and to identify predictive factors for controlled BP. In this study carried out in 2014, 280 patients were included consecutively through sampling from both university and private medical centers/pharmacies in four Iranian cities. Demographic data as well as information about duration of HTN and prescribed medications, admission to emergency department (ED) because of HTN crisis, comorbidities, and control of HTN during the last 6 months by a healthcare provider were gathered. Adherence to anti-hypertensives was also determined using the validated Persian version of the 8-item Morisky Medication Adherence Scale (MMAS-8). Controlled BP was defined as systolic BP< 140 and diastolic BP< 90 mmHg in non-diabetics and < 130/80 mmHg in diabetics. Of 280 patients, 122 subjects (43.6%) had controlled BP. Among 55 diabetics, only two patients (3.6%) had controlled BP. Multiple logistic regression revealed the following variables as significant predictors of controlled BP: higher MMAS-8 score (adjusted odds ratio (OR)= 1.19, P= 0.03), fewer number of comorbid conditions (adjusted OR= 0.71, P = 0.03), having occupation as clerk/military personnel (adjusted OR= 1.03, P= 0.04), and not having history of ED admission during the last 6 months because of HTN crisis (adjusted OR= 2.11, P= 0.01). Considerable number of the studied patients had uncontrolled BP. Regarding the dramatic consequences of uncontrolled high BP in long term, it is advisable that careful attention by health care providers to the aforementioned factors could raise the likelihood of achieving controlled BP.

## 1. Introduction

Hypertension is a well-documented and significant risk factor for cardiovascular diseases. It has a high prevalence in both developed and developing societies and is estimated to be the cause of mortality in 12.8% of the all number of deaths (World Health Organization, WHO, 2015).

In recent years, various pharmacologic classes of anti-hypertensives have been introduced to control high blood pressure (BP). Despite the privilege of access to such invaluable medications, concerning reports still are made from different countries about considerable number of patients whose BP values do not meet the defined BP goals. For instance, in the US, only 52% of patients with hypertension have controlled BP (Low, Pelter, Deamer, & Burchette, 2015). This figure (i.e., controlled hypertension) has been reported as 11.8% in China (Cai, Liu, Zhang, Li, & Wang, 2012), 27.2% in South Korea (Lee et al., 2010), 37% in Saudi Arabia (Saeed et al., 2011), and 11.2% in Portugal (De Macedo et al., 2007).

There are limited reports from Iran about the state of controlled hypertension. In a former study in Isfahan, Iran, it was found that only 37.2% had controlled BP. They also reported that body mass index (BMI) of more than 25 kg/m^2^ had the greatest effect on uncontrolled state of BP, especially in male patients (Arabzadeh et al., 2014). In another study in the city of Isfahan, it was found that BP of the patients was within the target limit in just 20.9% of the patients. Here, factors such as older age and educational level of 6 to12 years of study were recognized as variables which had significant association with controlled BP (Gharipour et al., 2013). Ebrahimi et al. (2010), in a similar way, reported that just 35.1% of Iranian patients had controlled BP.

One of the issues that have gained attention in management of chronic non-communicable diseases is adherence of the patients to medications administered for them. There are several methods to assess adherence of the patients, among which standardized and validated questionnaires are more acceptable by the authors. The importance of such adherence determination scales in hypertensive patients is that by administering them to the patients, useful information will be yielded about prediction of controlled BP in this population (Oliveira-Filho, Barreto-Filho, Neves, & Lyra Junior, 2012).

Here, we decided to determine the rate of controlled BP and associated factors among a sample of Iranian hypertensive patients. We tried to improve the quality of the study by two methods. First, as described later, the patients were recruited from different medical settings. Second, in addition to demographic and hypertension-related factors, their adherence to prescribed medications was assessed. By recognizing the associated factors effective on controlled BP of the patients, we believe that monitoring such patients will be more amenable in long-term and achieving goal BP will be made easier.

## 2. Materials and Methods

In this study with cross-sectional design, we studied 280 known hypertensive patients aged more than 18 years with at least six months passed from the diagnosis and were being treated by one or more anti-high BP medication(s). The locations which were selected for sampling included cardiology outpatient clinic in a tertiary hospital affiliated to a university hospital, private cardiology and general practice offices, and pharmacy. These selected sampling centers were located in the cities of Tehran, Karaj, Kermanshah, and Bafgh.

When patients presented to the selected locations for follow-up of their condition, interviews were done by doctors and pharmacists of the research team. A pre-designed checklist was filled out. The variables of the checklist consisted of demographic data (age, gender, weight, height, occupation, educational level), HTN duration, medications prescribed for HTN, other comorbidities, the documents of admission to emergency departments because of HTN crisis, and control of hypertension during the last 6 months by a healthcare provider.

To determine the adherence to anti-hypertensives, the Persian version of the 8-item Morisky Medication Adherence Scale (MMAS-8) which was validated by our team in a previous study was administered to the patients (Moharamzad et al., 2015). The MMAS-8 was designed to assess how the patients adhere to their medications (Morisky & DiMatteo, 2011). In particular, in assessing the adherence of patients to take their anti-hypertensive medications, the MMAS-8 had a sensitivity of 93% in finding those individuals with poor adherence (Morisky, Ang, Krousel-Wood, & Ward, 2008). This scale was also demonstrated to be significantly associated with antihypertensive drug pharmacy refill adherence (Krousel-Wood et al., 2009). The overall score of this scale is from 0 to 8. Higher score reflects better adherence to the prescribed medications. In a previous study on 200 Iranian patients who were suffering from HTN, the Persian version of the MMAS-8 was reported to be valid and reliable tool with a Cronbach’s α coefficient of 0.697 and test–retest reliability of 0.940 (Moharamzad et al., 2015).

At the end, BP of the patients was measured. For this purpose, sphygmomanometer was used to measure BP on the left arm in seated position. The patients were asked to seat relaxed and not smoke for half an hour before BP measurement. Controlled BP (based on the guidelines recommended by the Joint National Committee on Prevention, Detection, Evaluation, and Treatment of High Blood pressure, JNC 7) was defined as systolic BP of < 140 mmHg and diastolic BP of < 90 mmHg. In diabetics, the systolic and diastolic BP values were considered under control at less than 130 and 80 mmHg, respectively (Chobanian et al., 2003).

### 2.1 Statistical Analyses

Descriptive indices like frequency and percentage were used to report categorical data; mean and its standard deviation (±SD) were used to express continuous data. In order to determine correlation between continuous variables and controlled BP, the Pearson’s correlation test was used.

The independent samples t-test was applied to compare the ratios of patients who had achieved controlled BP between two levels of each desirable dichotomous variable. In this test, the binary (two-level) variables were recoded as 0 and 1. Controlled or uncontrolled BP was also recoded as 0 and 1 to be suitable to be included in the analyses. Then, the ratios of patients who had controlled BP were compared between the two levels of each binary variable (i.e., mean of the variables was compared by the independent t test). Independent samples t test, as a parametric test is better than non-parametric Chi-squared test to find significant relationships, if any. Likewise, in order to determine the relationship between controlled BP and explanatory variables with more than two levels, analysis of variance (ANOVA) as well as the Duncan post hoc test was applied.

Finally, multiple logistic regression method was applied to find significant predictors of controlled BP. All analyses were done by the SPSS software for Windows (ver. 16.0). Significance level was set at 0.05. Type II error was considered as 20% (power of 80%).

### 2.2 Ethics

After describing the purpose of the study and gathering information, verbal consent was obtained from the patients.

## 3. Results

Of 280 studied patients, there were 162 females (57.9%). Mean (±SD) age of the total sample was 60.3 (±10.0) years. Many patients (204 subjects, 72.9%) were overweight/obese (BMI values of ≥ 25 kg/m^2^). There were 164 subjects (58.5%) with educational level of lower than high school diploma (i.e., junior school or illiterate). Regarding occupational status, there were 73 (26.1%), 41 (14.6%), 119 (42.5%), and 47 (16.8%) persons respectively in market/self-employed, clerk/military (there were only three military personnel; the rest were clerks), housewife, and retired/unemployment categories.

Mean (±SD) duration passed from HTN diagnosis and initiation of treatment was 7.23 (±5.97) years. About one-third of the patients (99 subjects, 35.3%) had documents indicated they were admitted to the emergency departments because of HTN crisis in the last six months. Most patients (82.1%, 230 cases) had been visited and BP checked during the preceding six-month period at least once by a health care provider. For 127 patients (45.3%), only one anti-hypertensive class had been prescribed and others were taking two or more than two medicines. Angiotensin receptor blockers (ARBs) were the most commonly used anti-hypertensive among the patients documented in one-fourth of the participants. This was followed by selective beta blockers in 10%, hydrochlorothiazide in 5%, and angiotensin receptor inhibitors in 3.6% of the patients. One-hundred and twenty patients (42.8%) had only HTN. But others had comorbidities. The most common ones were ischemic heart disease (IHD) with/without history of coronary artery bypass grafting (CABG)/percutaneous coronary intervention (PCI) seen in 65 cases (23.2%), followed by diabetes mellitus (DM) seen in 55 cases (19.6%), and dyslipidemia (51 cases, 18.2%). Other common comorbid conditions included chronic kidney disease (seven patients, 2.5%), hypothyroidism (four patients, 1.4%), psychiatric disorders (three patients, 1.1%), neurologic disorders (two cases, 0.7%), osteoarthritis (two cases, 0.7%), etc.

General practitioner (67 cases, 23.9%) and cardiologist (65 cases, 23.2%) were the most common presented physicians who patients reported that they visit to control their high BP. Mean (±SD) overall MMAS-8 score was 5.75 (±1.88).

According to definitions applied for controlled BP, 122 subjects (43.6%) had controlled BP. In diabetics, only two patients out of 55 diabetics (3.6%) had controlled BP.

Table 1 shows the Pearson’s correlation test results between continuous variables and controlled BP status. As depicted, only the MMAS-8 score, HTN duration, and number of comorbidities had significant relationship with controlled BP status. The association between the MMAS-8 score and controlled BP status was positive; so higher values of the MMAS-8 score increased the chance of controlled BP. For other two variables, on the other hand, the association was negative. In other words, with longer HTN duration and more comorbid conditions, the proportion (chance) of controlled BP decreased (i.e., negative correlation coefficients).

**Table 1:** The Pearson’s correlation test to determine associated factors with controlled blood pressure among 280 hypertensive patients

Variable	Correlation coefficient	P value
Age	- 0.067	0.264
MMAS-8 score	0.228	< 0.001
BMI (body mass index)	- 0.111	0.064
HTN duration	- 0.151	0.011
Number of anti-hypertensives*	- 0.079	0.186
Number of comorbidities	- 0.179	0.003

* Indicates the number of medications prescribed (about half of patients were receiving monotherapy and the rest were receiving combination therapy).

Table 2 depicts association between discrete variables and BP status. As seen, the proportion of patients with controlled BP was significantly different among levels of educational level and occupational categories. In order to consider these variables more carefully, we applied the Duncan’s post hoc test. The results showed that in the educational level, we had three different groups of controlled BP proportions. The groups consisted of high school diploma or lower level, those with B.Sc. or M.Sc. university degrees, and the third group was those with Doctorate or PhD degrees. Those with higher educational level were more successful to have controlled BP. For the “occupation” variable, we can only observe two groups where one group was clerk/military personnel and the other occupational group was those with other states (housewife, retired/unemployed, and being self-employed). As shown in Table 2, the first group had more chance to control their BP efficiently. No correlation was found between physician type and controlled BP.

**Table 2:** Analysis of variance (ANOVA) to determine association between discrete variables and controlled blood pressure among 280 hypertensive patients

variable	P value	Proportion of patients with controlled blood pressure in each group					
Educational level	< 0.001	Illiterate	Reading and writing	Junior high school	High school diploma	B.Sc./M.Sc.	Doctorate/PhD
		0.29	0.29	0.44	0.51	0.72	1.00
Occupation	< 0.001	Unemployed	Retired	Market	Housewife	Clerk/military	
		0	0.32	0.038	0.40	0.78	
Physician type*	0.137						

* There were four categories for physicians: general practitioner, internist, nephrologist, and cardiologist.

The results of the independent sample t-test are shown in Table 3. As observed, the patients who did not have HTN crisis in the last 6 months, those with no comorbid condition, being under insurance coverage, and those who did not have diabetes mellitus were more likely to have controlled BP.

**Table 3:** The independent samples t-test analysis to compare the ratio of patients who had controlled blood pressure in each level of dichotomous variables

		Controlled BP	P value
Gender	Male	0.45	0.530
	Female	0.42	
Control in the last 6 months	Yes	0.44	0.996
	No	0.44	
Anti-hypertensive therapy	Single therapy	0.50	0.065
	Combination therapy	0.39	
HTN crisis	Yes	0.25	< 0.001
	No	0.54	
Presence of comorbidity	Yes	0.38	0.019
	No	0.52	
Insurance coverage	Yes	0.41	0.008
	No	0.65	
Cigarette smoking	Yes	0.49	0.402
	No	0.42	
Diabetes	Yes	0.04	< 0.001
	No	0.53	
Dyslipidemia	Yes	0.43	0.945
	No	0.44	
Ischemic heart disease	Yes	0.37	0.214
	No	0.46	
Having other comorbidity	Yes	0.48	0.446
	No	0.42	

Table 4 represents the input variables recognized by the multiple regression model as statistically significant predictors of controlled BP status. Controlled BP was positively associated with higher MMAS-8 score and occupational category of clerk/military personnel. On the other hand, controlled BP was negatively associated with higher number of simultaneous comorbid conditions, and having been admitted to the emergency room during the last month because of HTN crisis.

**Table 4:** The input variables in the multiple logistic regression model recognized as significant predictors of controlled blood pressure

	B	SE	P value	OR
MMAS-8 score	0.17	0.08	0.03	1.19
Number of comorbidities	- 0.33	0.15	0.03	0.71
Clerk/military personnel occupation	0.03	0.54	0.04	1.03
No hypertension crisis in the last six months	0.74	0.30	0.01	2.11

Abbreviations: MMAS-8= 8-item Morisky Medication Adherence Scale; SE= standard error; OR= odds ratio.

## 4. Discussion

The obtained findings support significant association between the adherence level of the patients to the prescribed anti-hypertensives, number of comorbid conditions, having job as clerk/military personnel, and experiencing HTN crisis with controlled BP. The odds of having controlled BP in those with higher MMAS-8 score (reflecting better medication adherence) were 1.19 times of those with lower MMAS-8 score. Adherence level is an important issue and has gained much attention during recent years in control of chronic non-communicable diseases. The results of previous studies about high/good adherence in hypertensive patients report figures ranging from as low as 15.8% (Aşılar, Gözüm, Capık, & Morisky, 2014) to 58% (Krousel-Wood et al., 2009). The results we observed here are in agreement with a previous study. In a report on 223 hypertensive patients, the authors found that adherent patients were more likely (OR= 6.1) to have controlled BP (Oliveira-Filho et al., 2012). The MMAS-8 is a simple and self-employed 8-item scale which in a short time can yield useful information about adherence of the patients. Since this variable was one of the factors that had significant association with controlled BP, we recommend using this scale in clinical practice to assess patients with uncontrolled BP. So, adherence in such patients may be an issue which can be uncovered and managed appropriately.

Another factor which was found to be predictive of controlled state of BP was not having history of HTN crisis in the last six months which necessitated admission to emergency department of a hospital. Hypertensive crisis, which is defined as systolic BP ≥ 180 mmHg and/or diastolic BP ≥ 110 mmHg, can be either in the form of hypertensive urgency or hypertensive emergency. The latter one is associated with end organ damage (e.g., stroke, heart attack, etc.) (Rodriguez, Kumar, & De Caro, 2010). Most experts agree that exact cause of hypertensive crisis is not fully understood. It has been advocated that hypertensive crisis is relatively common and unfortunately no change has occurred in its incidence in the past two decades worldwide (van den Born et al., 2011). We think that the finding of association between HTN crisis and uncontrolled BP with a considerable odds ratio of 2.11 has two-fold importance. First, admission to emergency services because of HTN crisis could alert the health care provider that the odds of facing a patient with uncontrolled BP are higher. Secondly, more follow-ups may be required after a patient with HTN crisis is discharged, though this was not our major objective and other prospective studies are required to elaborate this claim. Another issue which requires clarification is the nature of association between HTN crisis and medication use. We were not able to answer the question that whether HTN crisis occurred as a result of non-adherence to anti-hypertensives or basically this complication was the result of inadequate BP control. In either case, based on the analyses, this factor has clinical and preventive importance.

Another concerning finding relates to presence and number of comorbid conditions. From public health view, it is not uncommon to visit patients with other comorbid diseases such as diabetes mellitus, dyslipidemia, and so on (Schmieder & Ruilope, 2008). Compatible with what we observed here, poor BP control has been described in the setting of simultaneous medical condition. For instance, it has been reported that among stroke survivors, 55.3% did not have controlled BP within the target limits (Kesarwani, Perez, Lopez, Wong, & Franklin, 2009). Likewise, in another report, the rate of controlled BP among adult Americans with diabetes mellitus, stroke, heart failure, and coronary artery disease (23.2%) was significantly lower than that of those without these comorbid conditions (49.3%) (Wong et al., 2007). Based on our findings, the controlled BP had the worst condition among diabetics. Only 2 patients out of 55 diabetic patients had controlled BP of less than systolic BP of 130 mmHg and diastolic BP of less than 80 mmHg recommended by the JNC7 (Chobanian et al., 2003). Since the presence of other cardiovascular diseases risk factors and comorbidities, beside HTN, significantly raises the possibility of hypertensive complications and cardiovascular diseases and their progression, we recommend paying more attention to medication regimen and BP control of patients with comorbidities.

According to the performed correlation tests, those with higher educational level and having occupation category of clerk or military personnel had better controlled BP. A significant proportion of the patients studied were housewives. This is, we believe, another variable that may merit to be considered in follow-up of housewives which are likely to have uncontrolled BP. We did not determine economic status of the patients.

Two former Iranian studies reported some factors to be predictive of BP control. In one study, older age (i.e., age more than 65 years) with OR of 2.64 for controlled HTN and married individuals with OR of 2.19 for controlled BP (Gharipour et al., 2013) were reported to be predictors of good BP control. In the other study, BMI of more than 25 kg/m^2^ with OR of 13.09, male gender with OR of 8.47, and inappropriate attitude towards HTN (Arabzadeh et al., 2014) were recognized as significant predictors of poor BP control. However, we did not find these factors to be significantly associated with the controlled BP state. This discrepancy could be attributed to different factors in the study population, design of the studies, and variables of interest examined. For instance, in one study (Gharipour et al., 2013) only socioeconomic factors including marital status, educational level, income, and occupation were studied. We did not include income and marital status in our checklist. Also, sampling methods was different between our study and other two studies. Here, as we did not have access to data of hypertensive patients, convenient method was employed in clinics and drugstores to access the patients. But in the two mentioned studies, multistage sampling was done in three central cities of Iran (i.e., Isfahan, Najfabad, and Arak).

Hypertension is a complex condition and various factors can affect the ability of healthcare workers as well as patients to control it ideally. Factors suggested in previous studies to affect blood pressure include dietary factors such as restricted sodium intake (Perez & Chang, 2014), dynamic aerobic exercise (Cornelissen & Smart, 2013), work stress (Vrijkotte, van Doornen, & de Geus, 2000), psychological factors (Gerin & James, 2010), and so on. Unfortunately we were not able to monitor all these factors. It would be of outmost usefulness to include such factors in the future studies with larger sample size.

## 5. Conclusion

Medication adherence level, history of HTN crisis, and any comorbid conditions should be addressed during routine follow-up of hypertensive patients to achieve better BP control.
